# A ROS-Responsive Polymer-Micelle Delivery System Designed for the Treatment of Drug-Induced Myocarditis

**DOI:** 10.3390/pharmaceutics18101261

**Published:** 2026-10-06

**Authors:** Zhikun Lai, Ziyang Wang, Yunzhe Lang, Binghua Chang, Mingyu Mao, Wenqi Lu, Jiahao Weng, Yan Shen, Qimao Feng, Xiaozhen Hu

**Affiliations:** 1 Department of Cardiology, Shanghai Municipal Hospital of Traditional Chinese Medicine, Shanghai University of Traditional Chinese Medicine, Shanghai 200071, China; 2 Department of Geriatrics, Shanghai Municipal Hospital of Traditional Chinese Medicine, Shanghai University of Traditional Chinese Medicine, Shanghai 200071, China; 3 State Key Laboratory of Natural Medicines, China Pharmaceutical University, Nanjing 210009, Chinashenyan1982081@126.com (Y.S.)

**Keywords:** self-assembled micelles, inflammation microenvironment, glycyrrhetinic acid, targeted drug delivery

## Abstract

**Background/Objectives:** Drug-induced myocarditis lacks targeted therapies. Current glucocorticoid and immunosuppressant treatments suffer from poor lesion accumulation and systemic toxicity. We aimed to develop a ROS-responsive self-assembled nanocarrier (GSP) for the controlled release of glycyrrhetinic acid (GA) to inflamed myocardial tissue. **Methods:** GSP was synthesized by conjugating GA and PEG via a thioketal linker. Micelles were characterized by NMR, DLS, and TEM. ROS-triggered drug release, cytotoxicity, cellular uptake, anti-inflammatory (TNF-α) and ROS-scavenging effects were evaluated in DOX-activated H9C2 cells. In vivo, biodistribution, echocardiography, inflammatory cytokines, and histopathology were assessed in an isoproterenol-induced mouse myocarditis model. **Results:** GSP exhibited ROS-dependent release (>80% GA within 48 h under 100 mM H_2_O_2_ vs. <20% without). In vitro, GSP significantly reduced TNF-α and intracellular ROS in activated cardiomyocytes compared to free GA or non-responsive controls. In vivo, GSP showed enhanced cardiac accumulation via the EPR effect and markedly improved left ventricular ejection fraction and fractional shortening, reduced serum TNF-α, IL-1β, and IL-6, and decreased collagen deposition, with good biocompatibility. **Conclusions:** The ROS-responsive GSP nanocarrier enables on-demand drug release in the inflamed myocardial microenvironment, effectively attenuating inflammation and improving cardiac function, representing a promising targeted strategy for drug-induced myocarditis.

## 1. Introduction

Myocarditis is an inflammatory disease of the heart muscle [1], and its incidence has been increasing year by year. Drug-induced myocardial injury is one of the important triggers of myocarditis. For example, antineoplastic drugs (such as doxorubicin and immune checkpoint inhibitors) and antipsychotic drugs can directly or indirectly induce myocardial inflammation. Some patients with chronic myocarditis may progress to dilated cardiomyopathy [1], ultimately leading to heart failure or myocardial fibrosis, posing a serious threat to human health and life safety [2,3,4,5]. This type of drug-induced myocarditis also shows an increasing incidence year by year, particularly prominent in young and middle-aged populations, with persistently high mortality and disability rates.

Current treatment primarily relies on glucocorticoids, immunosuppressants, and supportive care. However, there is a lack of targeted drug delivery methods to the lesion site [5]. As a result, drugs often distribute systemically, leading to low effective concentrations at the lesion, limited efficacy, and significant toxic side effects [6]. Therefore, a novel therapeutic strategy that enables precise drug delivery and reduces systemic adverse reactions is urgently needed.

ROS drive the progression from inflammation to fibrosis by activating pathways such as NF-κB and TGF-β/Smad, making ROS a central hub in myocarditis pathogenesis [7,8,9]. In the myocarditis microenvironment, a high-intensity reactive ROS storm is synergistically released by neutrophils, M1-type macrophages, and damaged cardiomyocytes, exhibiting a biphasic evolution pattern characterized by an acute-phase peak and chronic-phase persistence. This unique dynamic feature allows ROS-responsive nanocarriers to utilize endogenous ROS as a precise drug release switch, achieving stage-adaptive therapy that provides rapid anti-inflammatory effects in the acute phase and anti-fibrotic effects in the chronic phase [10,11,12]. ROS-responsive micelles achieve on-demand drug release in the high-ROS lesion microenvironment, significantly reducing off-target toxicity and prolonging cardiac retention time compared with conventional delivery systems [13,14,15,16]. However, current research faces several systemic gaps. ROS-sensitive chemical linkages are largely derived from tumor-oriented designs and have not been optimized for the ROS profiles of myocarditis with different etiologies. In addition, there is a lack of animal models that recapitulate the chronic progression of the disease. Developing smart micelles tailored to the specific ROS features of myocarditis therefore represents a key direction to overcome current therapeutic bottlenecks.

Glycyrrhetinic acid (GA), a hydrolyzed product of glycyrrhizic acid and a pentacyclic triterpenoid compound, exhibits broad anti-inflammatory, antioxidant, immunomodulatory, and cardiomyoprotective effects [17,18,19]. GA significantly suppresses the NF-κB inflammatory signaling pathway [20,21,22], reducing the expression of pro-inflammatory cytokines and adhesion molecules, which helps mitigate immune-mediated myocardial injury. Oxidative stress levels are markedly elevated in myocarditis lesions. GA enhances antioxidant enzyme activity and inhibits ROS accumulation [23,24], thereby protecting myocardial cell membrane structure and mitochondrial function. This not only alleviates acute-phase injury but may also reduce the risk of chronic myocardial fibrosis [4,25,26,27].

In this study, we developed a microenvironment-responsive self-assembling nanocarrier as a targeted drug delivery system for myocarditis. A thioketal (TK) linker was used as a ROS-cleavable moiety. Through chemical synthesis, glycyrrhetinic acid (GA) and the TK linker were conjugated to the polyethylene glycol (PEG) polymer chain, which subsequently self-assembles into micelles. After systemic administration, these micelles accumulate at myocarditis lesions via the enhanced permeability and retention (EPR) effect. The high-ROS microenvironment then triggers the specific release of GA for anti-inflammatory treatment and improved cardiac function. The nanocarrier circulates stably in the body and achieves precise drug release at the lesion site.

## 2. Materials and Methods

### 2.1. Materials

Dulbecco’s Modified Eagle Medium (DMEM), Fetal bovine serum (FBS), trypsin-EDTA (0.25%) solution, Penicillin-streptomycin solution, and Far-red Plasma Membrane Fluorescent Probe (DID) were purchased from Jiangsu KeyGEN BioTECH Co., Ltd. (Nanjing, China). GA, EDC, NHS, and DMAP were purchased from Aladdin Biochemical Technology Co., Ltd. (Shanghai, China). Mouse ELISA kits were obtained from Beijing Solarbio Science & Technology (Beijing, China).

### 2.2. Methods

#### 2.2.1. Derivatization of GA

**Figure 1:**
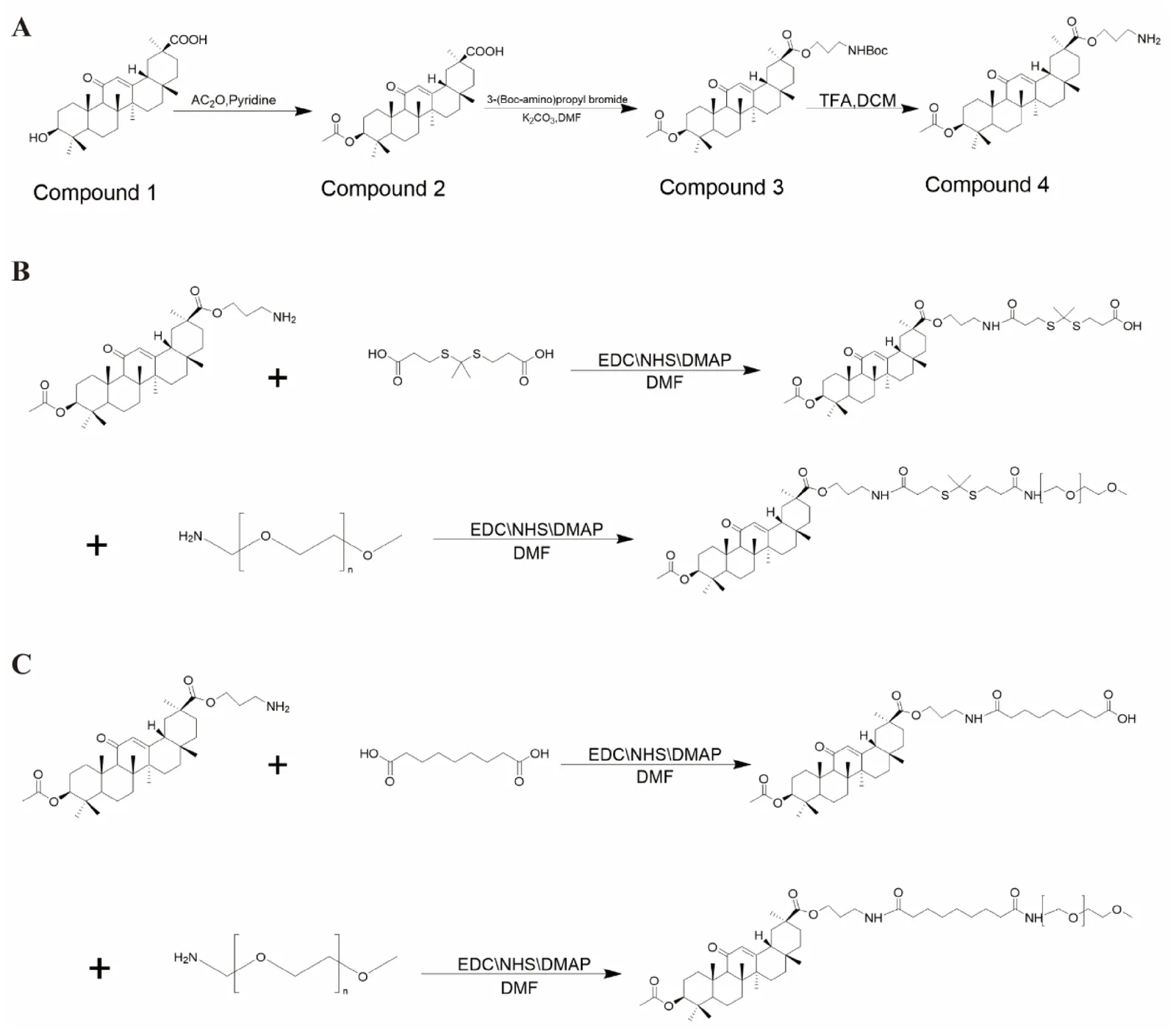
Synthetic routes of GA derivatives and polymers. (**A**) Amination reaction of GA to obtain GA-NH_2_. (**B**) Synthesis of ROS-responsive polymer GSP (GA-TK-PEG) via amidation. (**C**) Synthesis of non-responsive polymer GAP (GA-AZ-PEG) via amidation.

The reaction process for the amino derivatization of GA is shown in Figure 1A.

Preparation of Compound 2: Glycyrrhetinic acid (1.0 g) was accurately weighed and placed into a three-necked flask. The solid was dissolved in 6 mL of super-dry pyridine, which was measured with a syringe. Acetic anhydride (720 μL) was then added, and the mixture was stirred at room temperature while the reaction was monitored by TLC. After completion of the reaction, the reaction mixture was poured into water, and the pH was adjusted to 3–4 with hydrochloric acid. The crude product was washed with water, and the filter cake was washed with petroleum ether. The product was dried under vacuum to afford compound 2.

Preparation of Compound 3: A three-necked flask was charged with compound 2 (1.0 g) and anhydrous DMF (10 mL). K_2_CO_3_ (0.5 g), KI (325 mg), and 3-(Boc-amino)propyl reagent (0.6 g) were added successively, and the mixture was stirred at 50 °C overnight (TLC monitoring). After the reaction, the mixture was extracted with DCM, and the organic layer was washed with brine (3×), dried over Na_2_SO_4_, and concentrated in vacuo. The residue was purified by silica gel column chromatography to give compound 3 as a white solid.

Preparation of Compound 4: Compound 3 (1.0 g) was dissolved in DCM (10 mL) at 0 °C. TFA (2.5 mL) was added, and the solution was stirred for 3 h (TLC monitoring). After the reaction, the mixture was quenched with saturated NaHCO_3_ solution to pH 8–9, extracted with DCM, and the organic layer was washed with brine (3×), dried over Na_2_SO_4_, and concentrated in vacuo. The residue was purified by silica gel column chromatography to give compound 4 as a white solid.

#### 2.2.2. Synthesis of Polymers GSP

The target polymer reaction process is shown in Figure 1B. The synthesis of polymer GSP (GA-TK-PEG) comprises two steps: (1) amidation reaction between TK carboxyl groups and compound 4 amino groups; (2) amidation reaction between TK carboxyl groups and PEG amino groups. In brief, TK (1 mmol, 1 equivalent), EDC (1 mmol, 1 equivalent), NHS (1 mmol, 1 equivalent), and DMAP (0.1 mmol, 0.1 mol) were dissolved in anhydrous dimethylformamide (DMF). The mixture was stirred for 2 h under nitrogen to activate the TK carboxyl group. Compound 4 (1 mmol, 1 mol) was added to the solution, and the reaction proceeded at 250 rpm under nitrogen for 48 h. The reaction progress was monitored by thin-layer chromatography. Upon completion, the reaction mixture was extracted with dichloromethane. The organic layer was washed three times with saturated saline solution, dried over anhydrous Na_2_SO_4_, and concentrated under reduced pressure. The crude product was purified by silica gel column chromatography to afford the white solid compound 5. The carboxyl group of compound 5 underwent amidation with the amino group of PEG in the presence of EDC, NHS, and DMAP to synthesize GA-TK-PEG. Similarly, GA-TK, EDC, NHS, and DMAP (GA-TK conjugate:EDC:NHS:DMAP = 1:1:1:0.1, mol:mol:mol) were dissolved in anhydrous DMF. Under nitrogen atmosphere, the carboxylic acid of the GA-TK conjugate was activated by stirring for 2 h. After adding PEG, the mixture was stirred at 250 rpm for 48 h under nitrogen protection. The reaction solution was then placed in a dialysis bag (MWCO 1.0 kDa) and dialyzed against pure water for 48 h with frequent water exchanges. The dialyzed solution was centrifuged to remove water-insoluble byproducts, and the polymer GSP was obtained by freeze-drying.

The non-responsive polymer GAP (GA-AZ-PEG) reaction process is shown in Figure 1C.

#### 2.2.3. Characterization of GSP/GAP

The composition of the resulting GSP\GAP conjugate was confirmed by NMR spectroscopy (Brook Technology Co., Ltd., Beijing, China). Samples were dissolved in 10 mg·mL^−1^ deuterated chloroform for detection. Dissolve appropriate amounts of GA-NH_2_, TK, AZ, GSP, and GAP in DMSO. Perform full-wavelength scanning using a UV-visible spectrophotometer (Shanghai Meipuda Instrument Co., Ltd., Shanghai, China) to conduct UV-spectral analysis.

The critical micelle concentration (CMC) of GSP\GAP was determined using pyrene as a fluorescent probe. A series of polymer solutions with fixed pyrene concentration of 6.0 × 10^−7^ M was prepared, and fluorescence spectra (Shimadzu Experimental Equipment Co., Ltd., Shanghai, China) were recorded using a fluorometer (excitation wavelength: 337 nm). Emission fluorescence was detected within the wavelength range of 300–470 nm. The CMC was determined by calculating the ratio of emission intensities between the first peak (I_1_, 374 nm) and the third peak (I_3_, 385 nm).

The GA content in the micelles was determined by HPLC. Briefly, the freshly prepared GAP or GSP micelle solutions were centrifuged at 3500 rpm for 15 min at 4 °C to remove free GA. The supernatant was collected and lyophilized. The resulting powder was dissolved in the mobile phase consisting of acetonitrile and 2% acetic acid in water (80:20, *v*/*v*), filtered through a 0.22 μm membrane, and analyzed by HPLC (RP-C18 column, 4.6 × 250 mm; flow rate 1.0 mL/min; UV detection at 254 nm; injection volume 10 μL). The GA concentration was calculated from a calibration curve. The drug loading content (DL%) was calculated as follows:

$$\mathrm{Drug}\;\mathrm{loading}\;\mathrm{content}\;(\%)\;=\;\frac{Weight\;of\;the\;GA}{Weight\;of\;micelles}\times 100\%$$

#### 2.2.4. Preparation and Characterization of GSP/GAP Micelles

GSP micelles were prepared using the solvent diffusion method. Briefly, 10.0 mg of GSP conjugate was dissolved in 0.2 mL of ethanol, and the mixture was rapidly injected into 10.0 mL of deionized water with continuous stirring for 10 min. The mixture was then dialyzed against pure water in a dialysis bag (MWCO 1.0 kDa) for 6 h, with frequent replacement of the dialysis medium to remove ethanol. GAP micelles were prepared using the same method.

The particle size, polydispersity index (PDI), and zeta potential of the micelles were determined by dynamic light scattering (DLS) using a Zetasizer Nano ZS90 (Malvern Instruments, Great Malvern, UK). Measurements were performed at 25 °C with a scattering angle of 90°. Samples were diluted to a final polymer concentration of 0.5 mg/mL in deionized water prior to measurement. Each sample was measured in triplicate, and each measurement consisted of 10 runs. The reported mean hydrodynamic diameters represent intensity-weighted distributions. Zeta potentials were calculated from electrophoretic mobility using the Smoluchowski equation. Data are presented as mean ± SD (n = 3). Morphological changes were observed using transmission electron microscopy.

#### 2.2.5. ROS-Responsive Behavior of the TK Linker

To validate the ROS-responsive behavior, PBS solutions containing different concentrations of H_2_O_2_ (10 mM and 100 mM) were used. These concentrations were selected based on their widespread use in the literature for proof-of-concept evaluation of thioketal-based ROS-responsive systems. Prepare GSP micelle solutions and incubate them at 37 °C with constant agitation at 180 rpm for 12 h under conditions without H_2_O_2_, with 10 mM H_2_O_2_, and with 100 mM H_2_O_2_. Evaluate changes in micelle particle size using a particle size analyzer. Dispense several drops of the polymer solution onto a copper mesh coated with a carbon film. Perform counterstaining with 1% Phosphotungstic acid, allow to air-dry naturally, and then observe via electron microscopy.

To investigate the release profile of GSP under ROS conditions, GSP micelles were placed in hydrogen peroxide solutions of varying concentrations. Incubation was performed at 37 °C with constant agitation at 180 rpm for 48 h. The supernatant was separated using ultrafiltration centrifuge tubes with a cutoff of 3 kDa, and the released drug was analyzed by HPLC.

#### 2.2.6. Cytotoxicity Studies

The cytotoxicity of the formulation on H9C2 cells was assessed using the MTT assay. A series of formulation concentrations were prepared in basal medium for later use. H9C2 cells were cultured in 96-well plates and incubated at 37 °C in a 5% CO_2_ incubator for 24 h until cell confluence. After discarding the medium, 100 μL of medium containing different drug concentrations was added to each well. After 24 h of incubation, the medium was discarded, and 100 μL of 0.5 mg/mL MTT solution was added. The wells were then incubated for 4 h, after which the MTT solution was discarded. A total of 150 μL of DMSO was added to each well and the solution was shaken to dissolve any crystalline residue at the bottom. The absorbance was measured at 490 nm for each well using a microplate reader. The cell viability rate was calculated using the following formula:

$$\mathrm{Cell}\;\mathrm{viability}\;(\%)\;=\;\frac{A_{sample}-A_{blank}}{A_{control}-A_{blank}}\times 100\%$$

#### 2.2.7. Cellular Uptake

H9C2 cells were seeded in 6-well plates and cultured for 24 h before exposure to 0.5 mM DOX. After 8 h, DOX-activated H9C2 cells were incubated with the loaded DID formulation at 37 °C for 4 h, with free DID serving as the control. Different micelles can be prepared and incubated with 1 uM concentration of DID dye in the dark for 20 min to obtain DID-labeled micelles. After three PBS washes, cell uptake was observed using a laser scanning confocal microscope. Cells were suspended in PBS and analyzed by flow cytometry for fluorescence intensity, with untreated cells serving as the blank control.

#### 2.2.8. In Vitro Cell Culture and Treatment

Seed H9C2 cells in 6-well plates and culture for 24 h. Treat cells with DOX induction and various interventions (GA, GAP, GSP). Determine TNF-α levels in cells using the kit according to the instructions. Measure all samples in triplicate.

#### 2.2.9. ROS Clearance Capacity

Inoculate H9C2 cells into 6-well plates and culture for 24 h. Cells were treated with DOX induction and various interventions (GA, GAP, GSP). Intracellular ROS production was measured using 2’,7’-dichlorodihydrofluoroacetate (DCFH-DA). Imaging was performed under confocal microscopy, and quantification was conducted using a fluorometric microplate reader.

#### 2.2.10. Ethical Approval

All experiments were performed in accordance with the Chinese National Standard GB/T 35892–2018 (Guidance for the Care and Use of Laboratory Animals) and were approved by the Institutional Animal Care and Use Committee (IACUC) of China Pharmaceutical University (Approval No. YSL-202508036). Male ICR mice (6–8 weeks old, body weight 22–26 g) were purchased from the Jiangsu Qinglongshan Biotechnology Co., Ltd. (Nanjing, China). All animals were specific pathogen-free, had no genetic modifications, and had not undergone any previous procedures. Health status was confirmed by daily observation prior to the experiment. Mice were group-housed (5 per cage) in standard polypropylene cages with sterile wood-chip bedding, and provided with nesting paper and wooden blocks as enrichment. Humidity was maintained at 50 ± 10%. Animals were acclimatized for 1 week before experiments. Animals were monitored twice daily (morning and evening) for signs of distress (labored breathing, reduced mobility, hunched posture, weight loss > 20%). No adverse events (e.g., severe cyanosis or death) were observed. Humane endpoints were predefined: (i) inability to reach food/water, (ii) sustained weight loss > 25%, or (iii) severe lethargy; any mouse reaching these would be euthanized immediately by CO_2_ narcosis and cervical dislocation. No animals met these criteria. Analgesics were not administered because the ISO model does not cause surgical pain.

#### 2.2.11. Biodistribution of Micelles

The mice were fed in an environment controlled at 22 ± 1 °C, with light and dark cycles for 12 h and free diet and water. The experiment was carried out one week later. To investigate the distribution of the formulation in mice, DiD-labeled GSP micelles were administered intravenously. This study adhered to the internationally accepted standards for animal research following the 3Rs principle. The ARRIVE guidelines were used to report experiments involving live animals and to promote ethical research practices. In order to study the metabolic patterns of drugs, we first injected GA, GAP, and GSP into the tail vein of mice, at a dose of 0.1 mL/10 g, with the same GA dose in each group (20 mg/kg). Then, blood samples were collected from the eye socket at 0 h, 1 h, 3 h, 6 h, 12 h, and 24 h, and acetonitrile was added to precipitate the plasma protein in the tissue homogenate. The mixture was vortexed for 1 min and centrifuged at 10,000 rpm for 10 min. The entire supernatant was aspirated and filtered through a 0.22 μm microporous membrane for HPLC analysis of drug content. Groups (n = 5): GA, GAP, and GSP.

Then inject DID, DID@GAP, and DID@GSP into the tail vein of mice; administer a DID dose of 0.3 mg/kg via micro intravenous injection to each group of mice. Using a live imaging device to analyze the fluorescence intensity of the heart at 1 h, 2 h, 4 h, and 8 h, three mice in each group were euthanized at 4 h, and the main organs (heart, liver, spleen, lungs, and kidneys) were dissected. The main organs were washed with PBS, and residual blood stains were gently removed using filter paper strips. Fluorescence imaging of major organs was performed using the IVIS Lumina III (PerkinElmer Inc., Waltham, MA, USA) in vivo optical imaging system, and the fluorescence intensity was semi-quantitatively analyzed using Living Image software (Living Image 4.8.4) to obtain the average fluorescence intensity of myocardial injury hearts after different NDDS treatments. Groups (n = 8): DID, DID@GAP, and DID@GSP.

#### 2.2.12. Myocardial Injury and Treatment

Mouse Myocardial Injury Model: This experiment employed isoproterenol to induce a mouse myocarditis model. Low-dose isoproterenol (7.5 mg/kg bw) was administered via intraperitoneal injection for 10 consecutive days. Mice were observed for signs of diminished grip strength, including cyanosis of the lips, reduced food intake, slowed reactions, and lethargy. Serum cTnT levels were measured using a kit, and myocardial tissue underwent HE staining.

Mice were randomly assigned to groups and prepared as isoproterenol-induced myocarditis animal models per the aforementioned method. Following establishment of the myocarditis mouse model, each group received intravenous injections of different formulations (PBS, free GA, GAP, and GSP). Administration occurred every three days for a total of 14 days. Control groups included healthy (PBS only) and ISO-induced model groups. The experimental unit is a single mouse. Groups (n = 5): Control, PBS, GA, GAP, and GSP. Inject at a dose of 0.1 mL/10 g, with the same GA dose (20 mg/kg) for each mouse.

After treatment, hair loss was observed on the mice’s chests. Under 2% isoflurane anesthesia, transesophageal B-mode and two-dimensional M-mode images of the left ventricle were captured in mice via Doppler echocardiography. The system was employed to calculate the left ventricular ejection fraction (LVEF) and left ventricular shortening fraction (LVFS).

Evaluation of Left Ventricular Weight and Mass Index: Following cardiac scoring, the right ventricle is excised along the interventricular septum, excluding the septum itself. The excised tissue is weighed on a precision electronic scale (1/10,000 division) to obtain the left ventricular weight (LVW) in milligrams (mg). The left ventricular mass index (LVMI) is calculated as (LVW in mg) divided by mouse body weight (g), expressed in mg/g.

#### 2.2.13. Tissue Analysis and Immunohistochemistry

Cardiac tissue was fixed in 4% paraformaldehyde and embedded in paraffin. Masson’s trichrome staining was performed for analysis.

#### 2.2.14. Detection of Pro-Inflammatory Cytokines

Tumor necrosis factor-α (TNF-α) and interleukin-6 (IL-6) were detected as pro-inflammatory cytokines using ELISA kits according to the manufacturer’s instructions. All samples were measured in triplicate.

#### 2.2.15. In Vivo Safety Study

Normal mice were grouped and administered the respective formulations via tail vein injection. For 7 days post-administration, daily monitoring of growth status and body weight measurement per mouse were conducted. Body weight change curves were plotted to evaluate the effects of each formulation on mouse growth, feeding, and activity.

The mice were euthanized by CO_2_ narcosis, and major organs were dissected. After thorough washing with PBS, the specimens were immersed in 4% paraformaldehyde solution and stored at room temperature. Paraffin embedding and histopathological sectioning were performed following H&E staining. Pathological conditions of tissues and organs in each group were analyzed to determine whether inflammatory reactions or other pathological states were present. Groups (n = 5): Control, GSP, GAP, and GA. Inject each group of formulations into mice via tail vein injection (20 mg/kg, calculated as GA).

#### 2.2.16. Statistical Analysis

Statistical analysis was conducted using GraphPad Prism software (version 9.5.1; GraphPad Software, San Diego, CA, USA). Image processing followed standard guidelines using Image J (version 1.53t; NIH, Bethesda, MD, USA), Photoshop CS (version 8.0; Adobe Systems, San Jose, CA, USA), and Illustrator CS software (version 11.0; Adobe Systems, San Jose, CA, USA). Data are presented as mean ± SD and were analyzed using two-tailed Student’s *t*-test, one-way ANOVA, or two-way ANOVA as appropriate. Statistical significance was set at *p* < 0.05.

## 3. Results

### 3.1. ^1^H NMR Data and MS Data of Different Compounds

The acetylation of GA was confirmed by ^1^H NMR and ESI-MS analyses. The ^1^H NMR spectrum (500 MHz, CDCl_3_) showed a characteristic downfield shift of the H-3 carbinyl proton to δ 4.51, confirming the successful introduction of the acetoxy protecting group at the C-3 hydroxyl position, which was further supported by the appearance of a three-proton singlet at δ 2.04 corresponding to the acetyl methyl protons. The C-12 olefinic proton was observed as a singlet at δ 5.71, indicating that the double bond in the triterpene core remained intact under the reaction conditions. The methyl groups attached to the pentacyclic skeleton gave a cluster of singlets in the high-field region (δ 1.36, 1.22, 1.16 and 0.87), with the six-proton signal at δ 0.87 assigned to the two gem-dimethyl groups at C-4. The ESI-MS spectrum exhibited a parent ion peak at *m/z* 513 [M + H]^+^, consistent with the calculated molecular weight of C_32_H_48_O_5_. Collectively, these data confirmed the successful synthesis of the acetyl-protected intermediate without detectable epimerization at the C-18 position.

Compound 3, the Boc-protected amino intermediate, was characterized by ^1^H NMR and ESI-MS. The ^1^H NMR spectrum revealed successful introduction of the Boc-protected aminopropyl chain, evidenced by the appearance of a nine-proton singlet at δ 1.42 (-NHC(CH_3_)_3_) and a triplet at δ 3.18 (-NHCH_2_CH_2_CH_2_O-) integrating for two protons. The methylene protons adjacent to the oxygen of the linker appeared as a multiplet at δ 4.15, while the carbinyl proton H-3 of the GA skeleton remained downfield at δ 4.78, consistent with the retention of the acetoxy group. The characteristic olefinic proton H-12 appeared as a broad singlet at δ 5.62. The Boc protecting group was further confirmed by diagnostic signals at δ 1.42 and δ 3.18, and the overall integration ratios were in good agreement with the expected molecular composition. The ESI-MS spectrum showed a molecular ion peak at *m/z* 670.0 [M + H]^+^, which is in good agreement with the calculated value of 670.48 for the molecular formula C_40_H_63_NO_7_. All ^1^H NMR signals were properly assigned with correct integrations, confirming the successful synthesis of Compound 3. These data confirmed the successful conjugation of the Boc-protected linker to the GA core.

Upon removal of the Boc protecting group, the resulting amino-derivatized GA (Compound 4) was confirmed by ^1^H NMR and ESI-MS. The complete disappearance of the Boc proton signal at δ 1.42 served as the key indicator of successful deprotection. In parallel, a diagnostic downfield triplet appeared at δ 2.94 (-NHCH_2_CH_2_CH_2_O-), integrating for two protons, which correlated with the unmasked primary amine. The H-3 carbinyl proton of the GA core remained visible at δ 4.49, and the C-12 olefinic proton was maintained as a singlet at δ 5.62, confirming the structural integrity of the GA skeleton under the deprotection conditions (TFA/DCM). All other characteristic proton resonances of the triterpene skeleton were observed in the expected regions, with integration values fully consistent with the proposed molecular structure. The ESI-MS spectrum exhibited a parent ion peak at *m/z* 570 [M + H]^+^, in excellent agreement with the calculated molecular formula of C_35_H_55_NO_5_. Collectively, these data confirmed the successful preparation of the GA-NH_2_ intermediate, ready for subsequent conjugation reactions.

### 3.2. Synthesis and Characterization of GSP/GAP Conjugates

**Figure 2:**
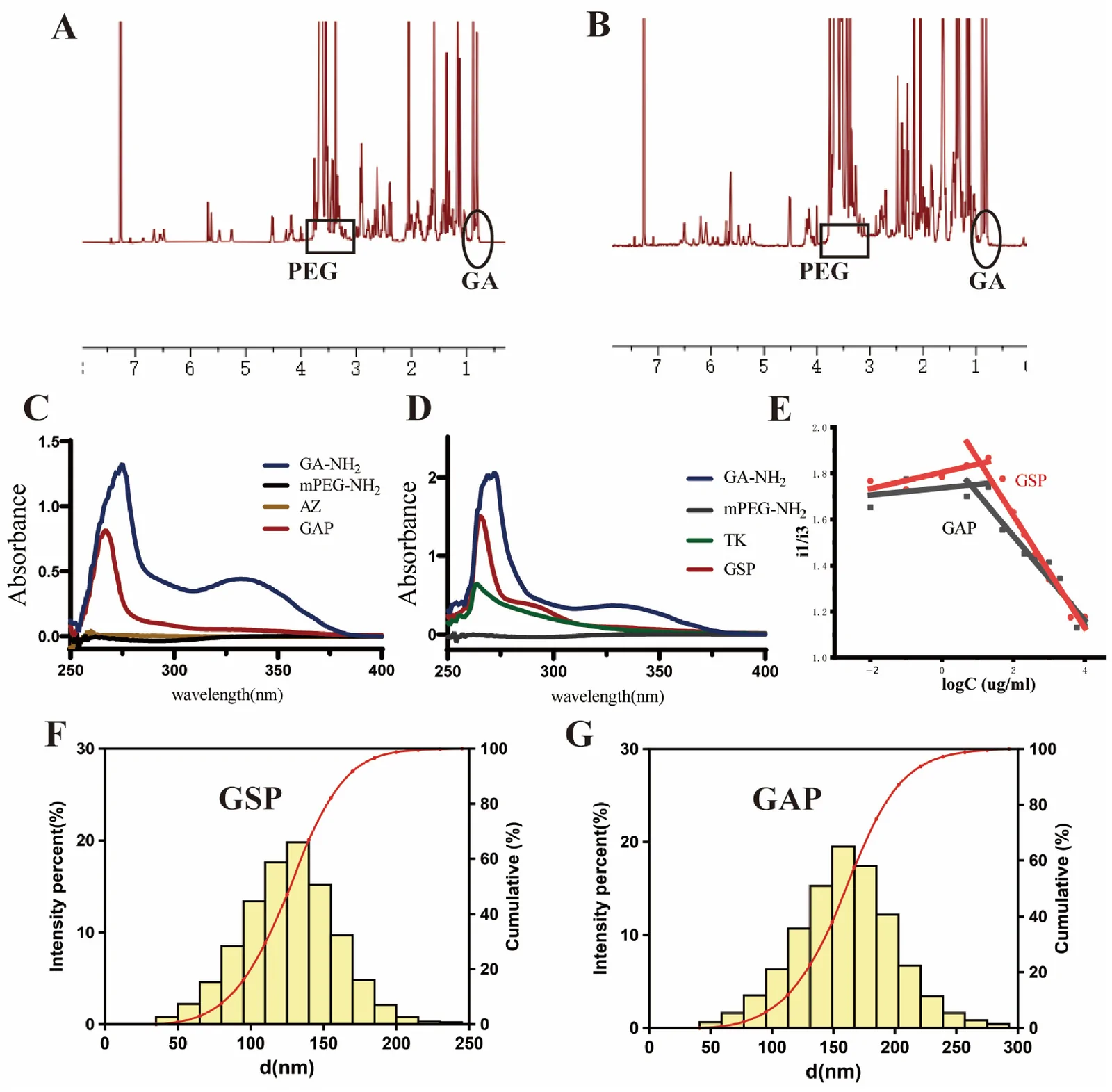
Characterization of GSP and GAP polymers. (**A**) ^1^H NMR spectrum of GSP in CDCl_3_. (**B**) ^1^H NMR spectrum of GAP in CDCl_3_. (**C**) UV-Vis spectra of GA-NH_2_, AZ, PEG, and GAP. (**D**) UV-Vis spectra of GA-NH_2_, TK, PEG, and GSP. (**E**) Determination of critical micelle concentration (CMC) using pyrene as a fluorescent probe. Plot of fluorescence intensity ratio (I_1_/I_3_) vs. logarithm of polymer concentration. Particle size distribution map of GSP (**F**) and GAP (**G**).

In the presence of EDC, NHS, and DMAP, the amidation reaction between the carboxyl group of TK and the amino groups of GA-NH_2_ and mPEG-NH_2_ successfully synthesized the polymer GSP (GA-TK-PEG). The detailed synthetic route is shown in Figure 1. The NMR spectrum was obtained in CDCl_3_, where micelle formation did not occur, and was used for structural confirmation of the polymer, with results shown in Figure 2A,B. The ^1^H NMR spectrum of GSP (Figure 2A) confirmed the successful conjugation of all three structural components. The PEG segment was identified by its characteristic strong multiplet at δ 3.5–3.7, corresponding to the repeating ethylene oxide units (-OCH_2_CH_2_-). The GA moiety was confirmed by its typical triterpenoid proton signals: the C-12 olefinic proton at δ 5.62 (singlet), the C-3 carbinyl proton at δ 4.51, and multiple methyl singlets of the pentacyclic skeleton in the high-field region between δ 0.8 and 1.4. The thioketal (TK) linker was identified by the methyl proton signal at δ 1.55 (singlet, 6H, -C(CH_3_)_2_-) and the methylene proton signals at δ 3.1–3.3 and δ 4.2–4.4. For GAP (Figure 2B), the spectrum exhibited characteristic signals of GA (δ 5.62, 4.51, and 0.8–1.4), the azobenzene (AZ) linker (aromatic protons at δ 7.2–7.8), and PEG (δ 3.5–3.7), confirming the successful synthesis of the non-responsive control polymer.

Different compounds selectively absorb light at specific wavelengths in the ultraviolet-visible region. Their absorption spectra exhibit structural specificity, enabling the identification of different species. Figure 2C,D shows the ultraviolet detection results of various compounds under full-wavelength scanning. Compounds mPEG-NH_2_ and AZ exhibit negligible UV absorption within the selected wavelength range. In contrast, compounds GA-NH_2_ and GAP display pronounced UV absorption between 200–300 nm with closely aligned absorption peaks, confirming the successful synthesis of GAP. Similarly, although compound TK exhibits an absorption peak in the 250–300 nm range, its absorption intensity is markedly lower than that of compounds GA-NH_2_ and GSP, confirming the successful synthesis of polymer GSP.

The inherent amphiphilicity of GAP/GSP polymers spontaneously forms micelles in water. The hydrophobic GA forms a hydrophobic core, while PEG forms a hydrophilic shell, moderately enhancing micelle stability in aqueous solutions. The CMC of the amphiphilic materials was determined using pyrene as a fluorescent probe. The CMC values for GSP and GAP conjugates were 39.7 and 79.4 µg/mL, respectively. These low CMC values ensure the conjugates form core-shell structured micelles in aqueous media while maintaining integrity and stability during systemic circulation.

### 3.3. Preparation and Characterization of GSP/GAP Micelles

The GSP and GAP micelles were successfully prepared via the solvent diffusion method. The micelle solution was dialyzed (MWCO 1.0 kDa) against pure water with frequent change of dialysis media to remove ethanol. Table 1 presents the particle size distribution and zeta potential distribution measured by dynamic light scattering (DLS), while the figure illustrates the morphology of micelles observed by transmission electron microscopy (TEM). The results indicated that GSP and GAP micelles exhibited uniform spherical shapes and were well-dispersed as individual nanoparticles.

The drug-loading percentages (DL%) of GSP and GAP micelles were 15.77 ± 1.21% and 15.78 ± 0.12%, respectively. The representative DLS size distribution curves for GSP and GAP are shown in Figure 2F,G, confirming monomodal and relatively narrow distributions for both formulations.

### 3.4. ROS-Responsive Behavior of the TK Linker

**Figure 3:**
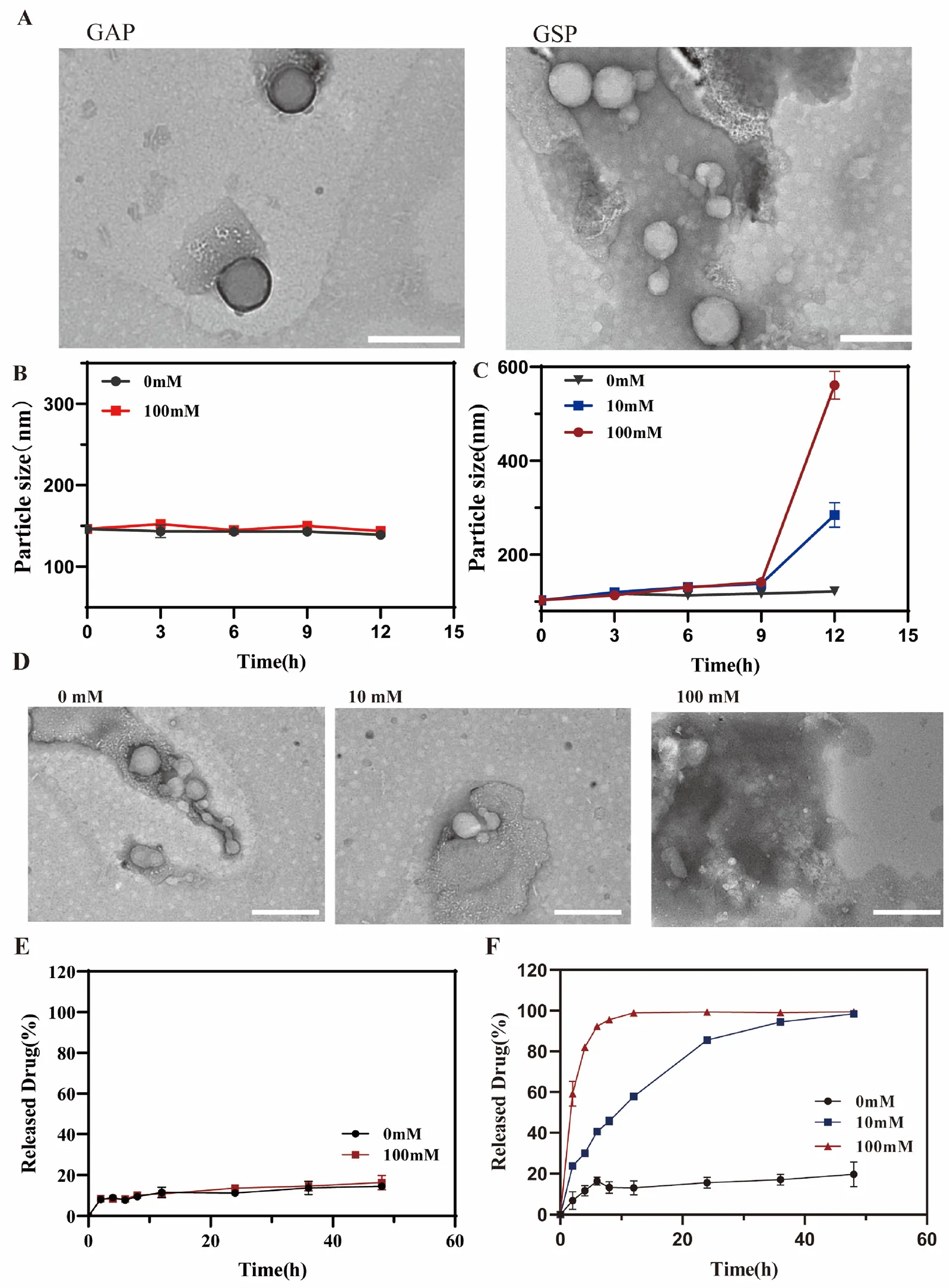
Characterization of ROS-responsive behavior of GSP and GAP micelles. (**A**) TEM images of GSP and GAP micelles. Scale bar: 200 nm. (**B**) Hydrodynamic diameter of GAP micelles under different ROS conditions measured by DLS. (**C**) Hydrodynamic diameter of GSP micelles under different ROS conditions measured by DLS. (**D**) TEM images of GSP micelles under different ROS conditions. Scale bar: 200 nm. (**E**) Cumulative drug release profiles of GAP micelles under different ROS conditions. (**F**) Cumulative drug release profiles of GSP micelles under different ROS concentrations.

The variation in size distribution of NPs under different ROS environments was used to evaluate the ROS-responsive behavior of GSP. Changes in the average particle size of GAP and GSP under different conditions and time points are shown in Figure 3B,C. Under conditions without H_2_O_2_ and at 10 mM, the GSP micelle particle size showed little change, indicating that the presence of H_2_O_2_ did not affect micelle stability. However, under high-concentration hydrogen peroxide conditions, the thioether bonds were disrupted, affecting the GSP structure and causing a sharp increase in particle size. Concurrently, transmission electron microscopy (TEM) observations of GSP micelle morphology under different conditions are presented in Figure 3D. The figure revealed that micelles remained uniformly distributed with intact cores in the absence of H_2_O_2_. At 10 mM, micelle edges exhibited slight blurring, while at 100 mM, GSP micelle edges were indistinct, the core-shell structure was disrupted, and micelles aggregated with each other.

Upon entering an inflammatory environment, the polymeric micelles rapidly release the encapsulated drug. In vitro release experiments employed H_2_O_2_ as a ROS trigger to investigate the carrier’s responsiveness. The in vitro release profiles of GSP and GAP at different hydrogen peroxide concentrations are shown in Figure 3E,F. Without hydrogen peroxide, GSP exhibited slow drug release, with less than 20% released within 48 h. In the presence of 10 mM hydrogen peroxide, nearly 60% of the drug was released within 12 h, reaching 80% release within 24 h. Under 100 mM hydrogen peroxide conditions, 80% of the drug was released within 4 h, and 90% within 8 h. In contrast, GAP exhibited extremely slow release at 100 mM hydrogen peroxide, showing no difference compared to the H_2_O_2_-free condition. These in vitro release experiments demonstrated that our prodrug carrier is a ROS-sensitive and specifically responsive drug delivery system that avoids burst release and leakage, properties that are ideal for reducing side effects and enhancing therapeutic efficacy.

### 3.5. In Vitro Cytotoxicity Assay of GSP/GAP Micelles

**Figure 4:**
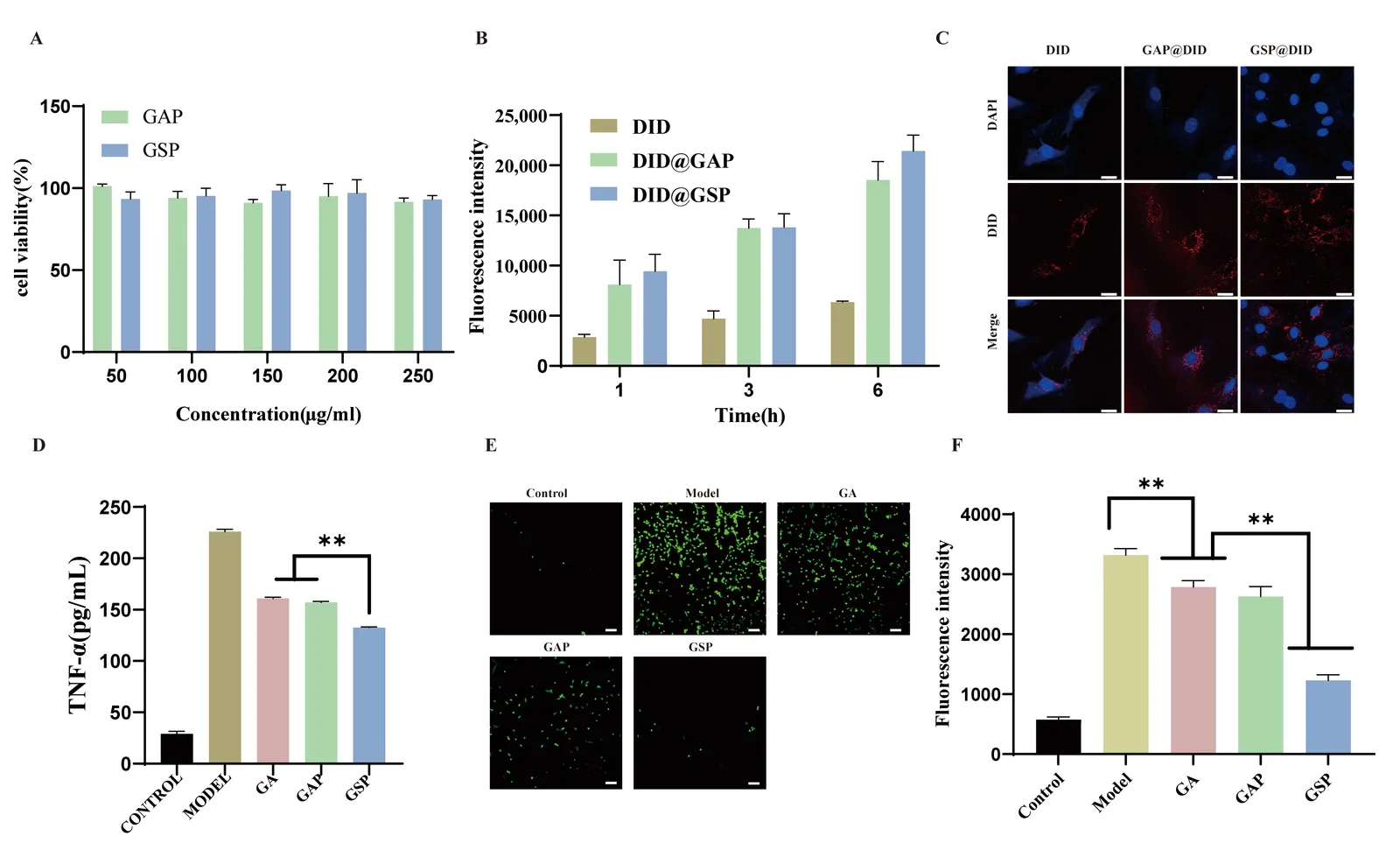
In vitro cytotoxicity, cellular uptake, anti-inflammatory activity, and ROS scavenging capacity. (**A**) Cytotoxicity of GSP and GAP micelles on H9C2 cells measured by MTT assay after 24 h of incubation. (**B**) Time-dependent uptake of free DID, GAP@DID, and GSP@DID by DOX-activated H9C2 cells quantified by flow cytometry. (**C**) Confocal microscopy images of cellular uptake after 6 h of incubation. Blue: DAPI-stained nuclei; red: DID fluorescence. Scale bar: 20 μm. (**D**) TNF-α levels in DOX-activated H9C2 cells after treatment with GA, GAP, and GSP. (**E**) Intracellular ROS levels measured by DCFH-DA fluorescence staining. (**F**) Quantitative analysis of ROS-associated fluorescence intensity. Data are presented as mean ± SD (n = 3). Statistical significance was determined by one-way ANOVA followed by Tukey’s post-hoc test. ** *p* < 0.01.

The cytotoxicity of each formulation was evaluated using the MTT assay. Results are shown in Figure 4A. When GSP and GAP concentrations ranged from 50 to 250 μg/mL, cell survival rates exceeded 90% after 24-h co-incubation with H9C2 cells within each group. No significant differences were observed between groups, indicating that within this concentration range, all nanoparticle carriers had no effect on cell survival rates and demonstrated good safety profiles.

### 3.6. Cellular Uptake Study

To investigate cellular uptake, H9C2 cells were treated with fluorescently labeled GSP, GAP, or free DID. In H9C2 cells, the internalization of free DID and micelles was analyzed using confocal imaging systems and flow cytometry. As shown in Figure 4B, we observed that uptake increased with prolonged exposure time for all formulations, indicating a time-dependent uptake pattern. Compared to free drug, NPs induced greater fluorescence intensity within cells, indicating strong uptake capacity for both nanocarriers. Unlike free drugs entering cells via passive diffusion, nanoparticle formulations entered cells in large numbers through continuous endocytosis and subsequently released the drug. Laser confocal images capturing uptake at 6 h are shown in Figure 4C. Blue fluorescence indicated DAPI-stained nuclei, while red fluorescence represents DID, serving as a surrogate marker for intracellular uptake. At this concentration, the fluorescent probe exhibited limited staining capacity, rendering free fluorescence negligible. When co-incubated with damaged cardiomyocytes, the GSP and GAP micelles exhibited significantly higher fluorescence intensity than the free group. The distribution of the formulation around the cell nuclei indicates successful uptake by activated cardiomyocytes. Endocytosis mediated by the EPR effect suggests strong uptake capacity of H9C2 cells for both formulations, consistent with flow cytometry results.

### 3.7. In Vitro Anti-Inflammatory Activity

The in vitro anti-inflammatory capacity of GSP micelles in DOX-induced H9C2 cells was investigated. DOX-activated H9C2 cells were treated with GSP micelles or other appropriate drug interventions. Cytokine TNF-α was measured via ELISA. Results shown in Figure 4D indicated that GSP micelles significantly ameliorated DOX-induced TNF-α production compared to DOX alone. Reduced TNF-α levels were also observed in H9C2 cells treated with free GA or GAP, reaching approximately 82% of the model group. This effect was primarily attributed to GA’s anti-inflammatory activity. Free GA passively diffuses into damaged cardiomyocytes with limited penetration, while GA exerts anti-inflammatory effects by inhibiting pro-inflammatory signaling pathways such as NF-κB, thereby reducing inflammatory cytokine production. Damaged cardiomyocytes internalized the nanocarriers GSP and GAP via endocytosis. Upon cellular entry, the non-responsive carrier slowly released GA, yielding therapeutic effects indistinguishable from the free acid group, with inflammatory factors reduced to 0.80 times the model group level. However, treatment with the responsive carrier GSP decreased inflammatory markers to 0.60 times the model group level, demonstrating significantly superior efficacy compared to GAP. This superiority is attributed to the presence of abundant ROS in the cellular inflammatory microenvironment, which sensitively triggers the responsive cleavage of GSP micelles, enabling rapid release of anti-inflammatory drugs for highly efficient anti-inflammatory effects.

### 3.8. ROS Clearance Capacity

Intracellular ROS levels were measured using the fluorescent probe DCFH-DA to evaluate the ROS scavenging capacity of different formulations in the inflammatory microenvironment. Results shown in Figure 4E,F indicated that when cardiomyocytes were damaged, cells produced large amounts of ROS. Compared to the model group, the intracellular fluorescence intensity was reduced in both the GA free drug group and the GAP micelle group, reaching 0.83 times that of the model group. This demonstrates that GA and GAP possess certain effects in reducing ROS levels and alleviating oxidative stress within cardiomyocytes. However, the limited passive diffusion into cells resulted in restricted anti-inflammatory effects. In contrast, the GSP group exhibited a marked decrease in intracellular fluorescence intensity, reaching 0.37 times that of the model group. This demonstrates that GSP rapidly undergoes bond cleavage under ROS conditions, releasing the active compound GA to exert antioxidant effects, thereby exhibiting strong ROS scavenging capacity against ROS generated by damaged cells.

### 3.9. Biodistribution of GSP\GAP Micelles

**Figure 5:**
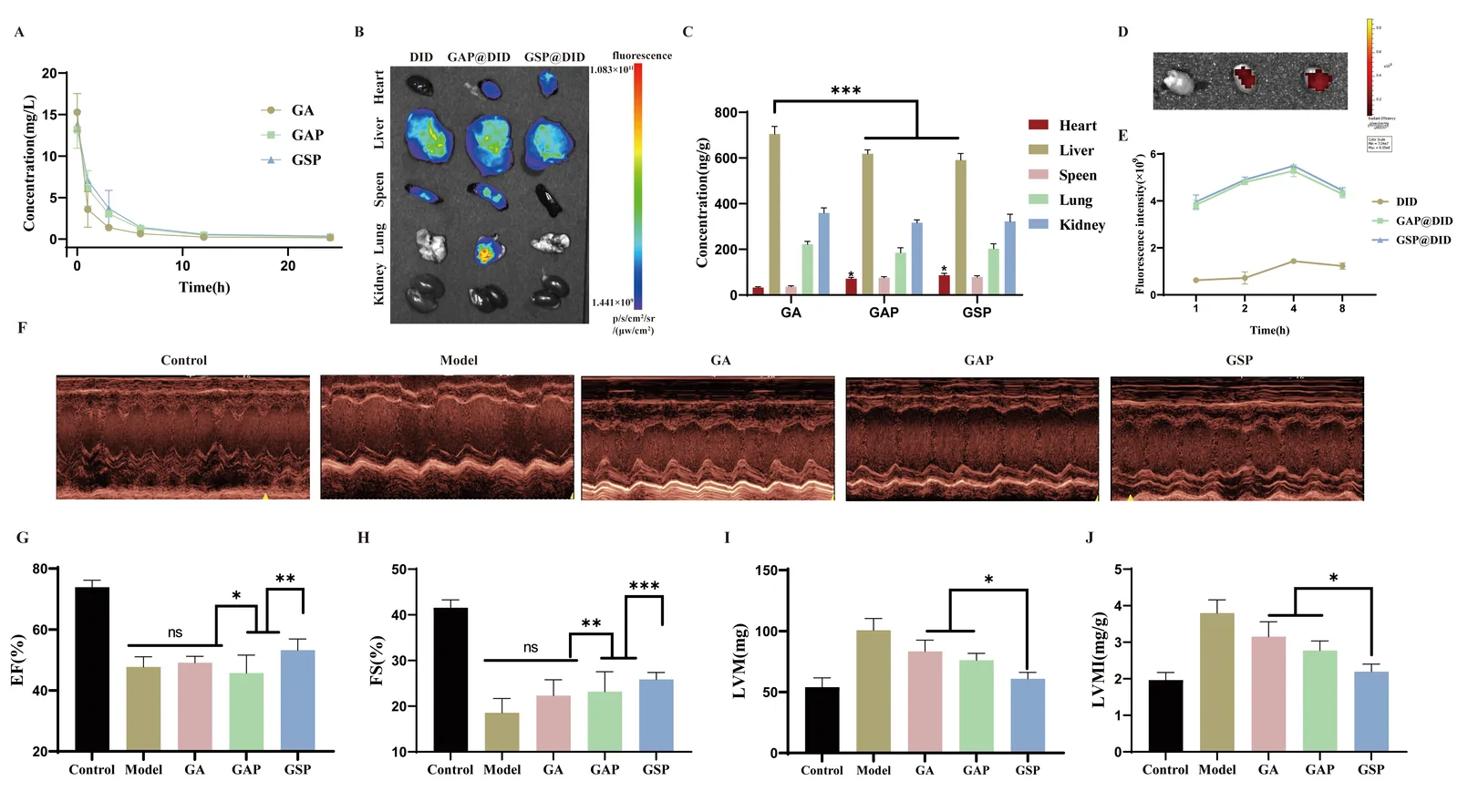
Biodistribution and in vivo therapeutic efficacy of GSP and GAP micelles. (**A**) Plasma concentration-time profiles of GA, GAP, and GSP after intravenous administration. (**B**) Ex vivo fluorescence images of major organs 4 h post-injection. (**C**) Quantitative biodistribution analysis of DID, GAP@DID, and GSP@DID in different organs. (**D**) Fluorescence intensity of NPs in the heart at 4 h post-injection. (**E**) Time-dependent fluorescence intensity of NPs in the heart. (**F**) Representative M-mode echocardiographic images of mice after different treatments. (**G**) Left ventricular ejection fraction (EF%). (**H**) Left ventricular fractional shortening (FS%). (**I**) Left ventricular weight (LVM). (**J**) Left ventricular mass index (LVMI). Data are presented as mean ± SD (n = 3). Statistical significance was determined by one-way ANOVA followed by Tukey’s post-hoc test. * *p* < 0.05, ** *p* < 0.01, *** *p* < 0.001, ns *p* > 0.05.

As shown in Figure 5A, the plasma concentration-time curves of the three formulations exhibited significant differences. The elimination half-life was calculated based on the concentration at hour 0 as the maximum blood drug concentration. The drug concentration in the GA group decreased most rapidly, with an elimination half-life of only 3.63 h. In contrast, the elimination rates of the GAP and GSP groups were significantly slowed, with half-lives prolonged to 5.75 h and 6.07 h, respectively. Compared with the free drug GA, the prodrug-modified GAP and GSP both showed markedly prolonged retention time in the systemic circulation, indicating that the prodrug strategy successfully extended the circulation time of the free drug. These results provide direct experimental evidence for enhancing the bioavailability and long-acting efficacy of the drug.

Intravenous injections of various fluorescently labeled formulations were administered to mice with myocardial injury. After 4 h, the mice were euthanized, and major organs were examined for fluorescence, with results shown in Figure 5B,C. Compared to free DID, GSP and GAP micelles exhibited higher fluorescence signals in the heart. At the site of myocardial injury, multiple inflammatory mediators stimulate contraction of capillary venule endothelial cells, enlarging intercellular gaps. This allows GSP and GAP micelles, which cannot pass through normal vascular walls, to passively and selectively leak from the bloodstream into the inflammatory tissue interstices. Leveraging the passive targeting of nanocarriers (EPR effect), drugs are precisely delivered to inflamed myocardial tissue. This enhances local drug concentration to maximize therapeutic efficacy while reducing systemic toxicity. However, a considerable proportion of the nanoparticles were distributed in the liver and spleen, similar to what has been observed for other nanoparticles. Analysis of the fluorescence intensity in the heart at different time points (Figure 5D,E) showed that the intensity first increased and then decreased, a trend consistent with the biological pattern of nanoparticles initially accumulating in vivo and then being metabolized.

### 3.10. In Vivo Efficacy of GSP\GAP Micelles in Myocardial Injury in Mice Model

Mice were intraperitoneally injected with isoproterenol to induce myocardial injury and then treated with different formulations. After two weeks of continuous administration, therapeutic efficacy was evaluated. At the end of the two-week treatment period, cardiac function was assessed in each group of mice using echocardiography. Results are shown in Figure 5F. Compared to the free drug group and the non-responsive vehicle group, the ROS-responsive formulation significantly improved cardiac function in mice, markedly enhancing contractile capacity. Analysis of M-mode echocardiography data yielded the ejection fraction (EF%) and Fractional Shortening (FS%) for each group, as shown in Figure 5G,H. Compared to the model group, neither the free drug group nor the non-responsive carrier group showed significant differences. The free drug distributed throughout the body upon administration but failed to accumulate at the inflammatory site to exert its therapeutic effect. In contrast, the non-responsive carrier retained at the inflammatory site via the EPR effect but could not rapidly release the drug after uptake by damaged cardiomyocytes, resulting in weak therapeutic efficacy. Following treatment with the responsive nanoparticle GSP, EF in mice significantly increased, rising by approximately 10% compared to the model group. The FS also increased by about 12% relative to the model group, with marked improvements in cardiac function indicators, highlighting the advantages of responsive nanoparticles. Similarly, GSP treatment reduced both left ventricular weight (LVM) and left ventricular mass index (LVMI) in mice (Figure 5I,J). Compared to free GA and GAP micelles, the responsive GSP micelles significantly decreased both LVM and LVMI. Left ventricular mass index serves as a standardized metric for assessing left ventricular myocardial function. GSP micelle treatment markedly improved myocardial function in mice.

**Figure 6:**
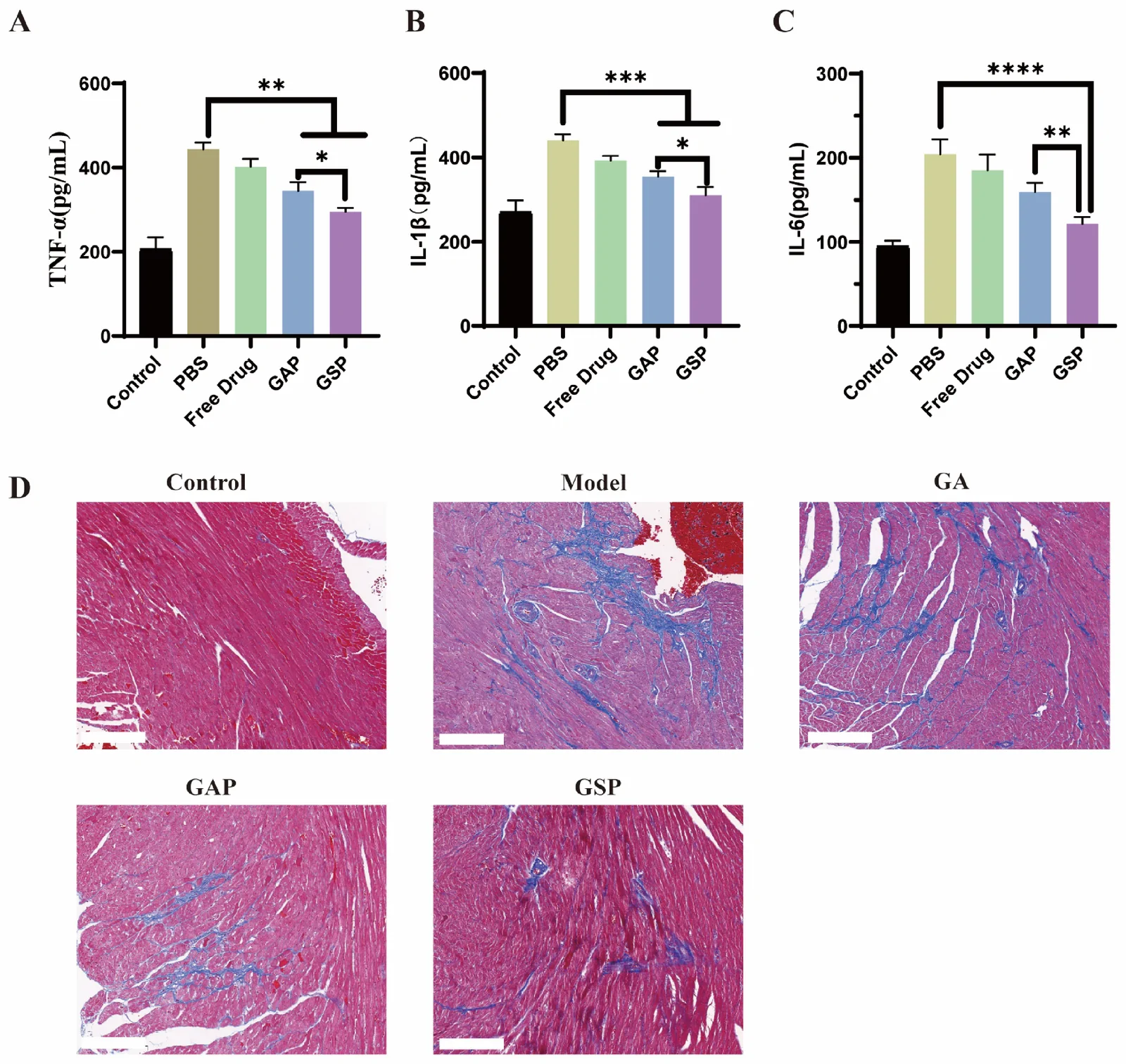
Anti-inflammatory effects of GSP and GAP micelles in ISO-induced myocarditis mouse model. (**A**) Serum TNF-α levels. (**B**) Serum IL-1β levels. (**C**) Serum IL-6 levels. (**D**) Representative Masson’s trichrome-stained heart sections. Blue staining indicates collagen deposition. Scale bar: 200 μm. Data are presented as mean ± SD (n = 5). Statistical significance was determined by one-way ANOVA followed by Tukey’s post-hoc test. * *p* < 0.05, ** *p* < 0.01, *** *p* < 0.001, **** *p* < 0.0001.

To evaluate the inflammatory response, the anti-inflammatory effects of GSP micelles were investigated in a mouse model of myocardial injury. Figure 6A–C showed that ISO treatment caused a sharp increase in pro-inflammatory cytokines TNF-α, IL-1β, and IL-6. Compared to the model group, animals treated with GSP micelles exhibited significantly reduced cytokine levels: inflammatory factor TNF-α decreased by approximately 33.6%, inflammatory factor IL-1β decreased by approximately 29.5%, and inflammatory factor IL-6 decreased by approximately 40.4%. Both GSP and GAP micelles accumulated and released drugs at the site of cardiac injury. However, GSP micelles exhibited specific release capability and sustained-release properties by rapidly releasing the anti-inflammatory drug GA in response to the high ROS characteristics of the inflammatory microenvironment, demonstrating superior anti-inflammatory effects.

Histopathological alterations serve as direct markers of cardiac dysfunction, indicating collagen deposition and inflammatory cell infiltration in the injured myocardium of mice. Masson’s trichrome staining was employed to evaluate collagen deposition in the damaged myocardial region. Compared to the model group, drug interventions (free GA, GAP, GSP) improved cardiac injury to varying degrees (Figure 6D). Among these, the responsive GSP micelle significantly reduced collagen deposition. Compared to free GA and GAP micelles, GSP micelles markedly ameliorated ISO-induced cardiac injury.

### 3.11. In Vivo Safety Study

**Figure 7:**
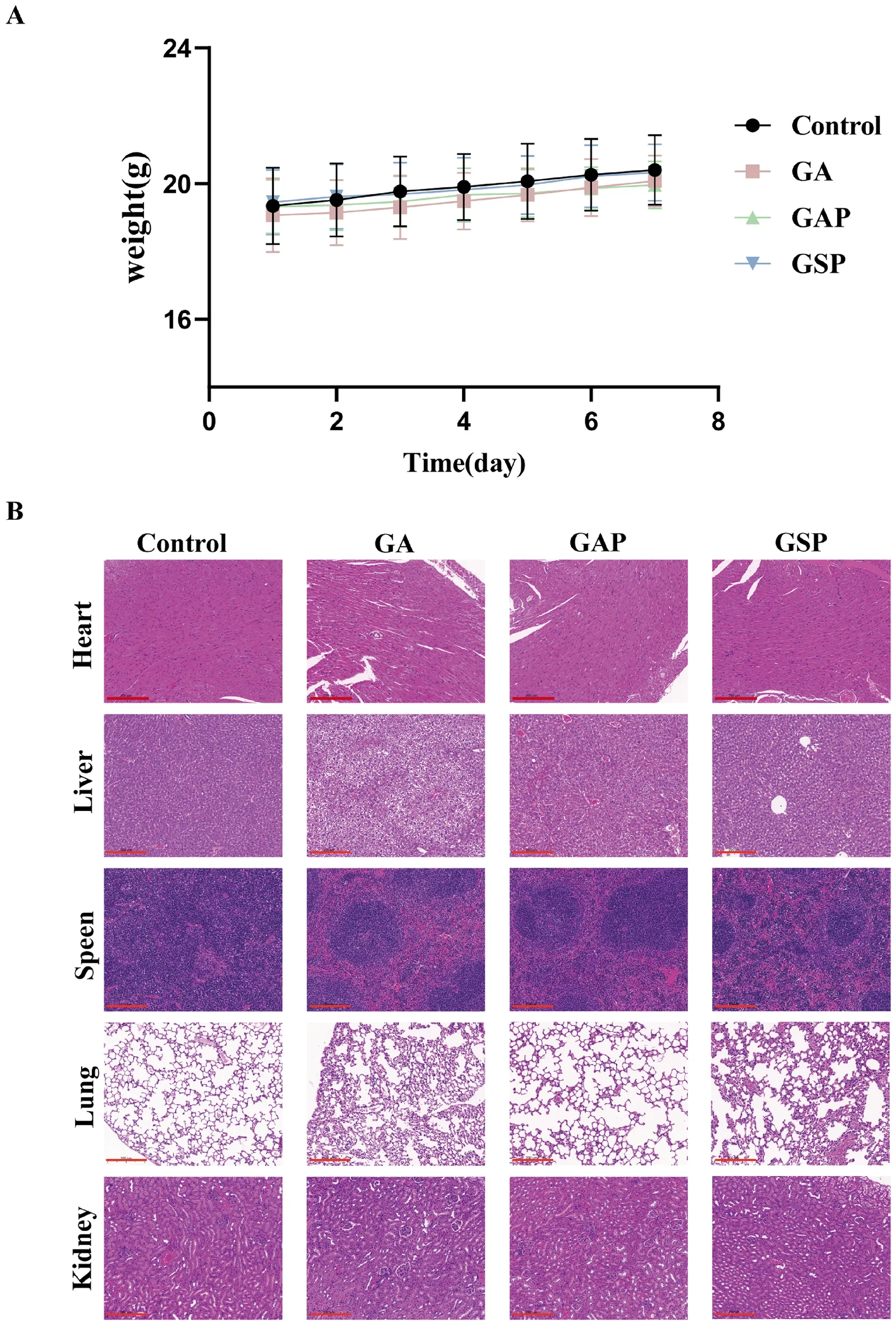
In vivo biocompatibility of GSP and GAP micelles. (**A**) Body weight changes of mice over 7 days after intravenous injection of PBS, free GA, GAP, and GSP. (**B**) Representative H&E-stained histological sections of major organs (heart, liver, spleen, lung, kidney) collected on day 7. Scale bar: 200 μm.

Under normal dietary and environmental conditions, mice received single intravenous injections of various formulations. Their activity levels and body weight changes were recorded, as shown in Figure 7A. Over seven days, mice exhibited normal feeding and urination patterns, maintained routine activity levels, and demonstrated good mental states. No significant redness, swelling, or hair loss was observed, and their appearance remained normal. Records of mouse body weight clearly showed a gradual upward trend. No significant differences in body weight were observed between the treatment groups and the control group.

HE staining of tissue sections is a common method for examining the pathological state of animal tissues and organs. Therefore, after administering different formulations to mice via intravenous injection, pathological sections of the mice’s major organs were obtained on the seventh day. The results were presented in Figure 7B. The mouse heart tissue exhibited clear striations, centrally located nuclei, no significant interstitial widening, and no inflammatory cell infiltration, indicating normal healthy heart tissue. Other organs showed no significant pigment deposition or inflammatory cell infiltration, consistent with normal histological sections. No obvious pathological changes such as necrosis or inflammation were observed in the major organs of mice administered any formulation group. This further confirmed that the prepared nanocarrier exhibits excellent in vivo biocompatibility and safety.

## 4. Discussion

In this study, we have successfully synthesized ROS-responsive polymers (GSP) as nanocarriers via an amidation reaction. Owing to their inherent amphiphilicity, they self-assemble into micelles in water. PEG coating and a low CMC confer excellent stability and prolonged circulation in vivo. Under normal physiological conditions, the responsive carrier released less than 20% within 48 h. However, in pathological environments containing ROS, the responsive GSP nanocarrier achieved over 80% release within 48 h, whereas the non-responsive nanocarrier released only 20%. This demonstrates a closed-loop system of “targeted localization-smart release-enhanced efficacy” for drug delivery, offering a novel approach for efficient and specific release of therapeutics in myocarditis treatment.

In vitro analysis of GSP demonstrated excellent biocompatibility. Cell uptake experiments confirm the nanocarrier’s time-dependent, efficient integration into cells via endocytosis. Upon entering damaged cardiomyocytes, the high intracellular ROS environment caused the polymer’s sulfide bonds to break, disassembling the polymer and efficiently releasing the anti-inflammatory drug GA. This inhibits cellular inflammatory progression, reducing pro-inflammatory factor levels and ROS concentrations. In vivo, the GSP nanocarrier passively targets damaged cardiac regions via the EPR effect, accumulates at injury sites, and exerts therapeutic effects. Echocardiographic analysis in mouse models reveals that the responsive GSP nanocarrier significantly improves cardiac function and reduces inflammation levels in vivo compared to the non-responsive GAP carrier. Furthermore, no systemic toxicity was observed with either GSP or GAP treatment, demonstrating favorable safety profiles.

Compared with previously reported ROS-sensitive cardiac delivery systems (e.g., TK-modified liposomes, TPP-targeted micelles, and phenylboronic ester-based hydrogels), our GSP system offers distinct advantages: a well-defined TK linker chemistry, stable self-assembly, high ROS-dependent release (>80% vs. <20% without H_2_O_2_), and the dual anti-inflammatory and antioxidant actions of GA. However, several limitations should be acknowledged. First, cardiac accumulation relies primarily on the passive EPR effect, which may be insufficient for detecting or treating early-stage or small inflammatory foci. Second, significant uptake in the liver and spleen was observed, indicating off-target distribution that could increase the risk of non-specific drug exposure. Third, the H_2_O_2_ concentrations used for trigger validation (10 and 100 mM) are supraphysiological; although they are commonly used for proof-of-concept, further studies using lower, pathologically relevant ROS levels are needed to confirm the in vivo sensitivity and responsiveness. Fourth, the current safety evaluation covers only 7 days, and longer-term chronic toxicity and repeat-dose safety remain to be assessed. These limitations highlight clear directions for future optimization, such as incorporating active targeting ligands to enhance cardiac specificity, reducing hepatic and splenic accumulation, and extending the safety monitoring period.

Our findings demonstrated that GSP is a microenvironment-responsive drug nanocarrier that senses the myocardial injury milieu and specifically responds to it, representing a promising delivery strategy for treating myocardial injury. Future research may focus on optimizing GSP’s specific accumulation and associated immunomodulation, as well as addressing the above-mentioned limitations to translate this system toward clinical application.

## Figures and Tables

**Table 1:** Physicochemical properties of GSP and GAP micelles. Data are presented as mean ± SD (n = 3).

Type of Micelles	dn (nm)	Zeta (mv)	DL (%)
GSP	136.92 ± 4.57	−17.77 ± 0.145	15.77 ± 1.21
GAP	159.78 ± 2.65	−15.76 ± 0.187	15.78 ± 0.12

## Data Availability

All the data are included within the manuscript.
